# Hidden Burdens: Psychological Distress Among Women Married to Military Personnel

**DOI:** 10.1192/j.eurpsy.2026.11928

**Published:** 2026-07-31

**Authors:** H. Schwartz, M. Kagan

**Affiliations:** Social Work, Ariel University, Ariel, Israel

## Abstract

**Introduction:**

Women married to career military personnel face distinctive challenges that combine the intensity of their spouses’ military service with gendered expectations of caregiving, family management, and emotional support. Frequent and prolonged absences, demanding routines, and repeated reintegration processes often generate role overload, loneliness, and marital tension, all of which may be associated with psychological distress. Despite their central role in family and community wellbeing, women married to career military personnel has received very limited academic attention.

**Objectives:**

The study examined how background variables (marriage duration, length of partner’s absence, presence of young children), psychosocial variables (role overload, loneliness, perceived social support), and marital variables (marital satisfaction, reintegration stress) contribute to psychological distress among women married to career military personnel.

**Methods:**

A cross-sectional online survey was conducted with 595 women married to career military personnel. Recruitment occurred via social media platforms. Participants completed standardized questionnaires and hierarchical regression model was used to test three main constructs: background, psychosocial, and marital.

**Results:**

Findings showed that psychosocial and marital variables explained a significant portion of psychological distress. Specifically, higher role overload, loneliness, and reintegration stress were strongly associated with greater psychological distress, while higher social support and marital satisfaction were linked to lower levels of psychological distress. Background variables showed no significant association with psychological distress.

**Conclusions:**

This study provides evidence from a large sample that women married to military personnel represent a vulnerable population facing elevated mental health risks. The findings contribute to theory by clarifying the interplay of background, psychosocial, and marital domains. The results highlight a neglected area requiring systematic attention from policymakers, health professionals, and the military system, with integration of this population’s needs into services to reduce distress and promote long-term wellbeing.

**Disclosure of Interest:**

None Declared

